# A psychologically informed, audiologist-delivered, manualised intervention for tinnitus: protocol for a randomised controlled feasibility trial (Tin Man study)

**DOI:** 10.1186/s40814-017-0137-8

**Published:** 2017-05-15

**Authors:** John A. Taylor, Deborah A. Hall, Dawn-Marie Walker, Mary McMurran, Amanda Casey, David Stockdale, Debbie Featherstone, Dean M. Thompson, Carol MacDonald, Derek J. Hoare

**Affiliations:** 1 National Institute for Health Research (NIHR) Nottingham Biomedical Research Centre, Ropewalk House, 113 The Ropewalk, Nottingham, NG1 5DU UK; 2 Otology and Hearing Group, Division of Clinical Neuroscience, School of Medicine, University of Nottingham, Nottingham, NG7 2UH UK; 3 Health Sciences, University of Southampton, Southampton, SO17 1BJ UK; 4 Division of Psychiatry and Applied Psychology, School of Medicine, University of Nottingham, Nottingham, NG7 2UH UK; 5 School of Life and Health Sciences, Aston University, Birmingham, B4 7ET UK; 6 British Tinnitus Association, Ground Floor, Unit 5, Acorn Business Park, Woodseats Close, Sheffield, S8 0TB UK; 7 Clitheroe Therapies Clinic, 3 Castlegate, Clitheroe, Lancashire BB7 1AZ UK; 8 Department of Psychology, University of Stirling, Stirling, FK9 4LA UK

**Keywords:** Tinnitus, Audiology, Psychological intervention, Randomised controlled trial (RCT), Manual

## Abstract

**Background:**

Chronic tinnitus is a common incurable condition often associated with depression, anxiety, insomnia and reduced quality of life. Within National Health Service (NHS) audiology in the United Kingdom (UK), no standard protocol currently exists for the treatment of tinnitus. Counselling is only available in less than half of audiology departments, and there is no agreed standard for what constitutes tinnitus counselling. There is substantial evidence from systematic reviews for the clinical benefit of cognitive behaviour therapy (CBT) for tinnitus delivered by clinical psychologists or psychiatrists, but no studies have sufficiently evidenced the NHS model of tinnitus care where management is increasingly being delivered by audiology professionals. In a pilot randomised controlled trial (RCT), this study aims to evaluate the feasibility of comparing a psychologically informed guidance manual developed to support audiologist management of tinnitus with usual treatment.

**Methods/design:**

Phase 1 consisted of three development stages: (1) a scoping review to generate a comprehensive set of tinnitus counselling components, (2) a Delphi survey involving expert patients (*n* = 18) and clinicians (*n* = 21) to establish consensus on the essential core attributes of tinnitus counselling, and (3) incorporation of these elements into a manualised care protocol. In phase 2, following training in a dedicated workshop, the manualised intervention will be delivered by three experienced audiologists across three different sites. Patients (*n* = 30) will be randomly allocated to receive either (1) psychologically informed management from an audiologist trained to deliver the manualised intervention or (2) treatment as usual (TAU) from an audiologist who has not received this training. Quantitative outcome measures will be administered at baseline, discharge and 6-month follow-up. Qualitative interviews with participating patients and clinicians will be conducted to gather perspectives on the feasibility and acceptability of the manualised intervention.

**Discussion:**

The feasibility of proceeding to a definitive RCT will be assessed via compliance with the manual, willingness to be randomised, number of eligible participants, rate of recruitment, retention and collection of quantitative outcome measures. This research offers an important first step to an evidence-based, standardised and accessible approach to tinnitus care.

**Trial registration:**

ISRCTN13059163. Date of registration: 6 May 2016

## Background

Chronic tinnitus is a common incurable condition where sound is perceived in the absence of acoustic stimulation. It affects about 10% of people in the United Kingdom (UK) [1] and is often associated with insomnia and hearing difficulty [2] and psychological problems such as depression and anxiety [3]. Within National Health Service (NHS) audiology, tinnitus is treated using a number of recommended interventions [4], but no standard protocol exists. As such, practices vary; sound therapy and patient education are widely available, but psychological interventions are only available in less than half of audiology departments [5].

A recent model of tinnitus posits that negative automatic thoughts and safety behaviours maintain tinnitus-related distress and that psychological interventions should be directed at reducing or removing these [6]. The benefit of cognitive behaviour therapy (CBT) delivered by clinical psychologists or psychiatrists is recognised [7–9], but there is no evidence for the effects of psychological interventions when delivered by audiologists [9]. Now that specialist tinnitus care is increasingly becoming the domain of audiologists due to the attrition of hearing therapists and the dearth of clinical psychologists in the UK, their delivery of a psychologically informed treatment represents a potentially cost-effective and highly accessible route to improving tinnitus patient care within the NHS. In their review of psychological interventions amongst tinnitus suffers, Wan Suhailah and colleagues [10] concluded that, due to the shortage of clinical psychologists, a simplified version of psychological intervention implementable by other clinicians should be developed to treat tinnitus. The Department of Health [4] also posit that audiologists should be able to provide psychological therapies where required. Furthermore, the effectiveness of audiologist-delivered CBT was one of the top 10 priority questions identified by clinicians and patients in the recent James Lind Alliance Tinnitus Priority Setting Partnership [11]. However, specialist audiological counselling skills are not a core requirement of the current audiologist training pathway in the UK, and in a survey, only 15% of audiologists in England considered themselves specialists in tinnitus care provision [5]. Although there is an appetite amongst audiologists to undertake further training in psychological therapies, the training is not standardised [12], nor has it undergone any formal evaluation. Thus, many audiologists have concerns about what degree of training is appropriate in order to formally ‘counsel’ [13]. This study represents a first step towards providing an evidence-base to support inclusion of psychological support for tinnitus into routine audiologist practice. In accordance with Medical Research Council (MRC) recommendations for developing and evaluating complex interventions [14], a phased approach to this study has been adopted, incorporating a development phase and an intervention phase.

The specific aims are:

1. To develop a treatment manual for a trained audiologist to use when providing psychological support for people with tinnitus
2. To conduct a feasibility trial to establish whether this manualised tinnitus treatment including core counselling elements versus treatment as usual (TAU) can be evaluated at the level of a large scale RCT
3. To conduct qualitative interviews with participating patients and audiologists to gather perspectives on the feasibility and acceptability of the manualised intervention

## Method

### Phase 1: development phase

This phase consisted of three stages:

1. *Scoping review:* components of psychological therapies for tinnitus were identified via a comprehensive scoping review of trials registers and academic databases of published literature, and editorials, conference abstracts and dissertations in the grey literature. A set of psychological therapy components were generated that underwent thematic analysis and were grouped across 25 themes [15].
2. *Delphi survey:* the set of psychological therapy components formed the basis of an online 3-round Delphi survey (Thompson et al., in preparation). The Delphi process is commonly used to develop consensus amongst experts when defining new interventions in health service research [16] and has been recognised as an appropriate method for this purpose [17]. The survey panel comprised patients (*n* = 18) who had experienced tinnitus counselling or psychological therapy and clinicians (*n* = 21) who had delivered counselling or psychological therapy for people with tinnitus, to establish consensus on the components of psychological therapy that audiologists could and should deliver in tinnitus management. Novel components elicited from the open-ended question in the first round were added to those generated from the scoping review. These 160 components were then rated in two subsequent rounds, where individual panellists were invited to reconsider their initial decisions in the light of overall levels of agreement across panellists. Consensus for inclusion or exclusion was taken as 80% or more of all panellists rating the component at points 6–7 or 1–2 on the scale, respectively. Consensus on the importance and relevance of 76 components was reached. For the remaining 84 components, consensus was not reached.
3. *Manualised protocol:* this stage comprised manualising the core components identified in the Delphi survey for testing in a feasibility trial as a specialised psychologically informed intervention delivered by audiologists. In order to describe and standardise the intervention, this stage commenced with a full day reference group meeting involving steering group and study team members to decide on which components to include and how to incorporate them into a psychologically informed manualised care protocol. It was agreed in this meeting that a working framework for the manual would comprise the following sections: rationale, assessment, education, treatment planning/goal setting, management/self-management and relapse prevention. All 76 components for which consensus for inclusion was reached were first considered for allocation under these headings. This was followed by considering whether any of the 84 components for which consensus was not reached should be included and if so, under which headings they would be written into the manual. Decisions were based on level of agreement from the Delphi survey, whether components had a good evidence-base, theoretical cohesiveness and what could realistically be included in a short, low intensity intervention delivered by audiologists, on the basis of resource limitations and time-limited training. Individual members of the team (including patient representatives, audiologist, hearing therapist, cognitive behaviour therapist and researchers) worked to develop specific sections of the manual in accordance with their areas of expertise and taking note of research into what makes a good treatment manual [18]. Finally, the manual was reviewed and amended to reflect a ‘whole’ in terms of style, coherence and theory.

### Phase 2: intervention phase

#### Feasibility trial design

**Fig. 1:**
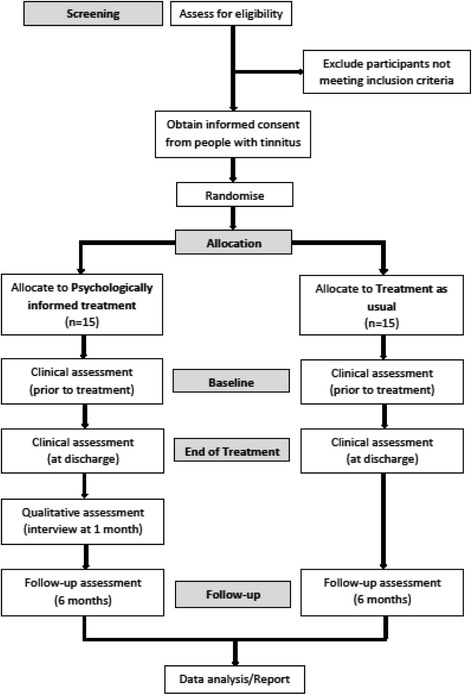
Psychologically informed treatment for tinnitus participant flow chart

The design is a non-blinded, multicentre, randomised controlled feasibility trial of psychologically informed treatment from an audiologist who has received training in the use of the manual, versus TAU from an audiologist who has not, for people with tinnitus (Fig. 1).

#### Participants

Thirty participants referred by a general practitioner (GP) or an ear, nose and throat (ENT) specialist for tinnitus management will be recruited. Recruitment will be across three NHS audiology departments which provide tinnitus services in Nottingham University Hospitals NHS Trust, Derby Teaching Hospitals NHS Foundation Trust and Sherwood Forest Hospitals NHS Foundation Trust. GP referral sample size was calculated to estimate critical parameters such as recruitment rate and acceptability of randomisation and different outcome measures. Following consultation with six clinical sites, it was estimated that, in general, recruitment of two participants per month per site was readily achievable. The sample size was therefore predicted rather than actual as it was determined by forecast, the number of sites and the timeframe of the project. It is not necessarily limited to the inclusion of just two participants per site per month. A predicted recruitment rate does however test the feasibility and likely timeframe of conducting a fully powered, multi-centre RCT. Furthermore, in order to estimate standard deviation, which can be used as part of a sample size calculation for an explanatory RCT, two authoritative papers taken together put the recommended estimated feasibility trial sample size in between 24 and 50 [19, 20].

##### Inclusion criteria

For inclusion in the feasibility trial, patients must reflect the typical population that might be expected to take part in a phase 2 clinical trial. The typical population includes adults aged 18 and over, comprises more males than females, shows a trend towards increasing age to a peak of between 60 and 69 years [21] and a bias towards people of higher deprivation/level of education [22]. The sample is also likely to contain a proportion of people who suffer from comorbid mental health conditions, e.g. anxiety and depression, and medical conditions, e.g. hypertension and diabetes mellitus [21]. The majority are also likely to have some degree of hearing loss [22]. Participants will be required to demonstrate the intellectual/cognitive capacity to provide informed consent and have sufficient mobility to attend clinics. No exclusions will be made on the basis of previous experience with psychological services. All patients will be enrolled at or before their first visit to audiology and have a primary complaint of tinnitus. Patients will also be required to present with a clinically relevant need, determined by a questionnaire score of >24 on the Tinnitus Functional Index (TFI) [23] indicating that tinnitus is at least a moderate problem. Offering this intervention to patients without such a clinically relevant need may be considered unnecessary treatment. All participants must be willing to complete questionnaires and to take part in follow-up semi-structured interviews about their experiences of taking part in the study.

##### Exclusion criteria

For the feasibility trial patients whose symptom of tinnitus is actually of a medically treatable origin (i.e. not chronic subjective tinnitus, but objective tinnitus for instance, where the noise can be detected by a third party) will be excluded. Also excluded will be patients who are unable to communicate in English and those participating in other research in tinnitus management at any time from consenting to their participation. Finally, patients who have comorbid conditions (e.g. psychotic disorders or dementia) which render them unable to give informed consent will be excluded. No exclusions will be made specifically on the basis of any medications patients may be taking.

### Ethical considerations

Ethical approval was granted by North West – Preston Research Ethics Committee (reference 16/NM/0047). Informed consent will be sought from participants in accordance with Good Clinical Practice (GCP) after thorough information about the study is provided by the researcher or site principal investigator (PI). The PI at each site will have responsibility for reporting of adverse events (AEs) to the chief investigator (CI) who will then inform the Research and Innovation Manager and Trust Governance Manager as required. Local policies and procedures at the three sites will be followed for reporting and investigating such incidents.

### Randomisation

Participants will be 1:1 randomised using the randomisation function in Microsoft Excel. First participants will be assigned a number between 1 and 10 for each of the three sites, according to chronological order of recruitment. For each site, these numbers will be given another random number using the Excel randomisation function and the participants 1 to 10 for each site will be ordered according to their associated randomly generated number. Those with the greatest five random numbers per site will be allocated to TAU. This process will be completed by telephoning a member of the research unit not involved in the trial after the participant has been screened as eligible and provided informed consent.

### Interventions

Psychologically informed treatment plus TAU: the intended purpose of the manual is to provide audiologists with a resource to support their work with people who have tinnitus in a psychologically informed therapeutic alliance to identify individual needs, set joint goals, reach shared and informed decisions and promote patient self-management. It is designed to go beyond the directive education and advice that might be considered TAU. Based on estimates from current practice, patients will participate in approximately five sessions, each lasting 1 h on average, delivered by an audiologist who attended a two-day training workshop on the manual.

The manual is divided into nine sections (Table 1). Section 1 provides the background and overview of the manual, including the processes and skills for developing a relationship with the patient. Section 2 involves assessment through a standardised interview, where the aim is for individual patient needs to be identified and a therapeutic alliance to begin. The rationale for psychologically informed treatment will then be discussed (section 3) prior to collaborative goal setting and treatment planning (section 4). Based on individual need, the audiologist and patient will work on relevant areas of tinnitus education (section 5) and management/self-management strategies (section 6). These include managing the emotional consequences of tinnitus, rapid relaxation, managing fear and avoidance behaviours, changing unhelpful thoughts and beliefs, promoting physical exercise, promoting good sleeping habits, attention, monitoring and acceptance, and sound therapy (enrichment). The treatment concludes with a focus on relapse prevention (section 7). Section 8 is a bibliography containing references related to the intervention and further reading for the audiologist or patient. The appendices (section 9) contain materials used for training in the manual. Audiologists will be guided on appropriate treatment areas to cover and resources to use from an accompanying ‘toolkit’ (Table 2). They will receive periodic peer supervision and from the manual trainers.

**Table 1:** Psychologically informed treatment for tinnitus manual contents

1. Introduction	
1.1.Background	
1.2.Overview of manual	
2. Patient assessment	
3. Rationale for psychologically informed treatment	
4. Goal setting/treatment planning	
5. Patient education	
6. Patient management/self-management	
6.1.Managing the emotional consequences of tinnitus	
6.2.Rapid relaxation	
6.3.Managing fear and avoidance behaviours	
6.4.Changing unhelpful (negative) thoughts and beliefs	
6.5.Promoting physical exercise	
6.6.Promoting good sleeping habits	
6.7.Attention, monitoring and acceptance	
6.8.Sound therapy (enrichment)	
7. Relapse prevention	
8. Bibliography	
9. Appendices	
A1.Communication in rehabilitation	
A2.The relational skills model—setting up the relationship	
A3.The relational skills model—developing the relationship	
A4.The relational skills model—working with the relationship	
A5.The relational skills model—the established relationship	
A6.Providing explanations	
A7.Teach-back technique

**Table 2:** Psychologically informed treatment for tinnitus toolkit

T1 Tinnitus functional index (and scoring rubric)	
T2 Tinnitus case history questionnaire	
T3 Formulation worksheet (for sections 2 and 6.3)	
T4 BTA leaflet all about tinnitus ver.1.4	
T5 Cognitive model of tinnitus psychology tool	
T6 Stages of change model	
T7 Goal setting sheet	
T8 Action planning sheet	
T9 Goal setting and action planning practice framework	
T10 What keeps tinnitus going psychology tool	
T11 What causes tinnitus psychology tool	
T12 Meaning in tinnitus psychology tool	
T13 BTA leaflet tinnitus and stress ver.1.4	
T14 Relaxation training diary	
T15 Fear and avoidance example formulation	
T16 Exposure worksheet	
T17 Negative thinking example formulation	
T18 Unhelpful thinking styles sheet	
T19 Thought record—blank copy	
T20 Thought record—example copy	
T21 Physical exercise example formulation	
T22 Physical exercise diary	
T23 Sleep cycles sheet	
T24 Sleep diary	
T25 BTA leaflet taming tinnitus ver.1.3	
T26 Acceptance in metaphors sheet	
T27 Mindfulness: three simple ways to get present sheet	
T28 My ‘managing tinnitus’ blueprint: making a plan for well-being	

Treatment as usual (TAU): patients will be assessed and managed by an audiologist who has not received training in psychological therapy and has not received training in the psychologically informed treatment for tinnitus. Based on routine clinical practice, this is likely to include management of tinnitus education and advice over approximately three sessions, each lasting 1 h on average.

### Outcome measures

Feasibility of a full-scale randomised controlled trial is the essential outcome of the feasibility trial. Feasibility for this would be confirmed if the feasibility trial demonstrates (1) a conservative recruitment rate of 10% of all eligible patients, i.e. screening 10 or fewer patients to gain a single recruit, (2) recruitment of 65% of target participants, i.e. recruiting 20 of the target number of 30, (3) retention of 80% of participants with an equivalent proportion of outcome data collected, and (4) continued patient and clinician compliance to the manualised tinnitus counselling.

Outcomes for the feasibility trial include completeness of a set of validated questionnaires. These reflect typical components of an RCT test battery in tinnitus and are considered most likely to capture the anticipated therapeutic benefits of the intervention:

1. Tinnitus Functional Index (TFI) [23] to measure the severity and impact of tinnitus
2. Tinnitus Cognitions Questionnaire (TCQ) [24] to assess positive and negative cognitions associated with tinnitus
3. Clinical Outcomes in Routine Evaluation - Outcome Measure (CORE-OM) [25] to assess general well-being
4. Health Utilities Index (HUI) 15Q [26] to assess health-related quality of life and cost-utility
5. Working Alliance Inventory (WAI) [27] to measure the quality of the therapeutic alliance (patient and clinician versions)
6. Section 4 of the Client Service Receipt Inventory (CSRI) [28] to measure the use of health and social care

The participant will complete the baseline study questionnaires in advance of their first audiology appointment and again immediately at the end of treatment (excluding the CSRI which is only completed at follow-up). To determine a candidate primary outcome measure of long-term impact, the questionnaires (including the CSRI) will be administered again via post at 6-month follow-up. One telephone or email reminder to complete the follow-up questionnaires will be used if the questionnaires have not been returned within 2 weeks.

### Qualitative interviews and focus groups

Clinicians delivering the manualised treatment and patients receiving it will be invited to partake in semi-structured interviews of approximately 1 h to discuss their experiences of the intervention and for treatment fidelity to be explored. Patient interviews will be carried out 4 weeks following their last appointment to capture continued compliance with treatment techniques whilst not compromising recall. Clinician interviews will be conducted after discharge of their final patient. The interviews will be audio-recorded and conducted by authors, JAT and DMT, transcribed and analysed using a thematic analysis approach [29]. Initial analysis of the qualitative data will be conducted by JAT and the study team, including members of the NIHR Nottingham Biomedical Research Centre Patient and Public Involvement (PPI) panel. Emerging themes will be discussed in two focus groups with (i) audiologists and (ii) patients, to ensure that the themes and any identified barriers and facilitators to treatment implementation and maintenance of self-management are accurately representative of patient and audiologist experience. The purpose of this outcome is to identify which components of the manualised care worked well, why and which elements were not useful. It is important to establish what was successful and unsuccessful since the intervention development started with patient and clinician opinion of what is important rather than based on which components of treatment are better than others.

### Statistical analysis

The feasibility trial will not include any hypothesis testing since its primary purpose is to determine whether a full-scale trial can be conducted. Descriptive statistics will be used to confirm (1) the number of eligible participants at each site (participating sites will routinely administer the TFI questionnaire to all patients presenting with a primary complaint of tinnitus), (2) willingness of patients to take part in a trial and be randomised (number of eligible participants willing to participate), (3) number of eligible participants recruited per month, (4) number of participants retained at discharge and follow-up, and (5) percentage completion rate of questionnaire outcome measures, including questions unsuccessfully and incorrectly completed.

Descriptive statistics, including standardised mean averages, standard deviation (SD), standard error and confidence intervals, will also be used to compare the evaluative and discriminative properties of the tinnitus specific outcome measures. This will provide data on a potentially best single measure to use, should this work proceed to an explanatory RCT. Responsiveness to change of the tinnitus questionnaires used will be compared by assessing change to standardised mean scores across assessment time points within patient groups that reported an improvement, worsening or no change in their tinnitus-related health.

## Discussion

Within NHS audiology, there is no standard protocol for the treatment of tinnitus. Although, research evidence highlights the benefit of CBT for tinnitus when delivered by psychologists or psychiatrists [7–9], psychological interventions are only available in fewer than half of audiology departments [5]. Furthermore, evidence for the effects of such treatments when delivered by audiologists is lacking [9].

With specialist tinnitus care becoming the domain of audiologists, a psychologically informed treatment is potentially an efficient, more effective means of their delivery of tinnitus care within the NHS. However, specialist audiological counselling skills are not currently integrated as a core requirement into the audiologist training pathway in the UK. Due to the lack of standardisation and evaluation of audiologist training in psychological therapies [12], many audiologists have concerns about their competence in this area [5, 13].

This feasibility trial will determine whether the manualised psychologically informed treatment developed for this study is appropriate, acceptable and practicable to patients and audiologists, and feasible to test in an exploratory trial. The feasibility trial will be used to inform any important changes to the care manual and inform the design of a trial to test its effectiveness. The aim of this study is to provide a platform for an integrated tinnitus counselling component as part of audiologist training as a first step towards inclusion of psychological support for tinnitus in routine audiologist practice.

## Abbreviations

AE: Adverse event
BTA: British Tinnitus Association
CBT: Cognitive behaviour therapy
CI: Chief investigator
CORE-OM: Clinical Outcomes in Routine Evaluation - Outcome Measure
CSRI: Client Service Receipt Inventory
ENT: Ear, nose and throat
GCP: Good clinical practice
GP: General practitioner
HUI: Health utilities index
MRC: Medical Research Council
NHS: National Health Service
PI: Principal investigator
PPI: Patient and public involvement
RCT: Randomised controlled trial
SD: Standard deviation
TAU: Treatment as usual
TCQ: Tinnitus Cognitions Questionnaire
TFI: Tinnitus Functional Index
UK: United Kingdom
WAI: Working Alliance Inventory
